# Blunted cardiac reactivity to psychological stress associated with higher trait anxiety: a study in peacekeepers

**DOI:** 10.1186/s12868-015-0216-9

**Published:** 2015-11-23

**Authors:** Gabriela Guerra Leal Souza, Ana Carolina Ferraz Mendonça-de-Souza, Antônio Fernando Araújo Duarte, Nastassja Lopes Fischer, Wanderson Fernandes Souza, Evandro Silva Freire Coutinho, Ivan Figueira, Eliane Volchan

**Affiliations:** Department of Biological Sciences, Federal University of Ouro Preto, Ouro Preto, MG Brazil; Forebrain Neurotecnologia Ltda., Rio de Janeiro, RJ Brazil; Brazilian Army Research Institute of Physical Fitness, Rio de Janeiro, RJ Brazil; Institute of Biophysics Carlos Chagas Filho, Federal University of Rio de Janeiro, Rio de Janeiro, RJ Brazil; Department of Psychology, Federal Rural University of Rio de Janeiro, Seropédica, RJ Brazil; National School of Public Health, Oswaldo Cruz Foundation, Rio de Janeiro, RJ Brazil; Institute of Psychiatry, Federal University of Rio de Janeiro, Rio de Janeiro, RJ Brazil

**Keywords:** Anxiety, Emotion, Cortisol, Heart rate, Psychological stress

## Abstract

**Background:**

Both exaggerated and diminished reactivity to stress can be maladaptive. Previous studies have shown that performing increasingly difficult tasks leads first to increased reactivity and then to a blunted response when success is impossible. Our aim was to investigate the influence of trait anxiety on cardiac and cortisol response to and recovery from a standardized psychosocial stress task (Trier Social Stress Task) in a homogeneous sample of healthy peacekeepers. We hypothesized that participants with higher trait anxiety would show blunted reactivity during the performance of an overwhelmingly difficult and stressful task. Participants (N = 50) delivered a speech and performed an arithmetic task in the presence of critical evaluators. Cortisol samples and electrocardiogram data were collected. Participants completed the State-Trait Anxiety Inventory—Trait version, the Posttraumatic Stress Disorder Checklist—Civilian Version (PCL-C) and the Military Peace Force Stressor Inventory.

**Results:**

For heart rate, the findings showed that peacekeepers with higher trait anxiety reacted less to the speech task (p = 0.03) and to the arithmetic task (p = 0.008) than those with lower trait anxiety. Trait anxiety did not modulate cortisol responses to the task. Despite the high trait anxiety group having higher PCL-C scores than the low trait anxiety group (p < 0.0001), this did not influence the cardiac results.

**Conclusions:**

We concluded that individuals with higher trait anxiety had less tachycardia in response to acute psychological stress than those with lower trait anxiety. The present results point to a higher risk for more anxious individuals of a maladaptive reaction to stressful events.

## Background

The reactivity hypothesis has been a key focus of research on the link between stress and health for several decades. It proposes that cardiovascular reactivity to psychological stressors, if prolonged or exaggerated, can promote poor future cardiovascular health [1–3]. Hypothalamus–pituitary–adrenocortical (HPA) axis reactivity to stress, one of the most common analysis in this field, has also been found to have a similar effect on cardiovascular health [4]. Given the emphasis placed on an association between exaggerated reactivity and disease pathogenesis, low or blunted reactivity to acute stress has been assumed to be benign or even protective. However, [5] it has been proposed that both extremes, exaggerated and diminished reactivity, could be maladaptive responses to stress and could promote allostatic overload. Indeed, recent evidence suggests that low or blunted reactivity to stress may actually have serious adverse consequences for health and behavior [6].

Trait anxiety refers to the existence of stable individual differences in the tendency to respond to stressful situations with state anxiety and in the anticipation of threatening situations [7]. Therefore, trait anxiety may play a key role in the calibration of the stress response system, insofar as it could promote a less adaptive response to stress that, in the long term, could induce an allostatic overload.

Carroll et al. [8], using a highly stressful psychosocial task, found that participants with higher levels of anxiety presented less cardiac reactivity. This study was considered the most relevant regarding trait anxiety outcomes in a recent meta-analysis [9], which indicated an association between trait anxiety and reduced cardiovascular reactivity with acute stress. However, of the 13 selected investigations, the one conducted by Carroll et al. [7] was unique in clearly showing blunted reactivity in highly anxious participants; the other studies either showed no differential reactivity [10–19] or even increased reactivity [20]. Notably, the acute stressor used in the study by Carroll et al. [7] was by far one of the most intense stressors in a laboratory setting; in that it was a difficult task performed in the presence of a critical evaluator. Interestingly, a study investigating the effect of task difficulty on the cardiovascular system revealed that pre-ejection period changes and systolic blood pressure reactivity increased until the task became so difficult that it was impossible to successfully complete. At this exact point, blunted cardiac reactivity was found [21]. Therefore, it could be hypothesized that highly anxious individuals may consider difficult and stressful tasks to be much more challenging than less anxious individuals do, therefore explaining why Carroll et al. [7] found more clearly blunted reactivity in subjects with high levels of anxiety.

When we look for evidence regarding the relationship between trait anxiety and cortisol response to a stressor, a marker of HPA-axis reactivity, the literature does not show a correlation between these two variables. To the best of our knowledge, no author has hitherto found consistent evidence about the impact of trait anxiety on cortisol response to acute stress. On the other hand, hypocortisolemia has been a feature extensively studied in patients with stress disorders like post-traumatic stress disorder, chronic fatigue, fibromyalgia and burnout [22]. Many of these disorders are commonly related to anxiety problems and therefore, it is also important to evaluate the impact of trait anxiety on cortisol response to an acute stress.

Given the mixed results found in the meta-analysis [9] regarding the acute physiological responses to laboratory-induced stress, and given the negative health outcomes associated with blunted reactivity to stress [6], it is important to elucidate the association of this reduced reactivity with high trait anxiety. A study design that could provide stronger outcomes and complementary answers to this research question should consider (i) studying a sample presenting minimal confounding factors and (ii) applying a strong stressor. In order to meet these criteria, the present investigation used a sample of peacekeepers to control for possible confounding variables. The sample was homogeneous in respect to gender, age, health and physical conditioning, allowing the study of trait anxiety and its association with cardiac reactivity to acute psychological stress to be relatively uncontaminated by other factors. Furthermore, we considered that the use of such a specific sample might also help to shed light on the relatively unexplored psychophysiology of a large range of professionals involved in risk-taking activities.

The stress protocol we used was the Trier Social Stress Test (TSST) [23], in which the participant is asked to deliver a speech followed by an arithmetic task in front of an evaluating audience and a camera. This task is considered one of the most demanding psychological stress tasks for humans [24]. This protocol consistently evokes subjective stress and activation of both the HPA and sympatho-adrenal-medullary system [25–28].

Therefore, the aim of this study was to investigate the association between trait anxiety and cortisol and cardiac reactivity to a standardized psychological stress task in a sample of healthy peacekeepers. We hypothesized that the task would be overwhelmingly difficult and stressful for participants with higher trait anxiety, leading to blunted cardiac and cortisol reactivity in those individuals.

## Results

Three participants were excluded from the cardiac analyses due to technical problems with the electrocardiogram recording, so the final sample comprised 47 participants. For cortisol, 21 participants were excluded due to technical problems, so the final sample comprised 29 participants. The mean PTSD Checklist—Civilian Version (PCL-C) score was 24.2 (SD = 6.02, range 17–40). No participant scored above the cut-offs that indicate a possible positive screen for PTSD (Veterans: cut-off = 50 [29], civilians: cut-off = 40 [30]), indicating that the peacekeepers in the present sample were mentally healthy in respect of this disorder. The mean score on the State-Trait Anxiety Inventory—Trait (STAI-T) was 34.2 (SD = 8.22, range 22–57). The mean score on the Military Peace Force Stressor Inventory (MPFSI) was 16.6 (SD = 10.83, range 0–46).

Student’s t test revealed significant difference between “low” and “high” trait anxiety groups in STAI-T (t = 8.98; p < 0.0001) and PCL-C (t = 4.73; p < 0.0001) (Table 1).

**Table 1:** Sample characteristics

	High trait anxiety N = 23	Low trait anxiety N = 24	T	p value
STAI-T	40.8 (6.30)	27.8 (3.23)	8.98	<0.0001
Age	25.3 (6.31)	25.9 (6.06)	−0.32	0.75
BMI	23.5 (2.45)	23.5 (2.87)	−0.02	0.99
Military service time	6.5 (6.46)	7.1 (6.23)	−0.34	0.73
Total of stressful events	18.6 (11.20)	14.3 (10.28)	1.16	0.16
PCL-C	27.7 (6.55)	20.8 (2.51)	4.73	<0.0001

*STAI-T* State-Trait Anxiety Inventory—Trait, *BMI* body mass index, *PCL-C* Posttraumatic Stress Disorder Checklist—Civilian Version

**Fig. 1:**
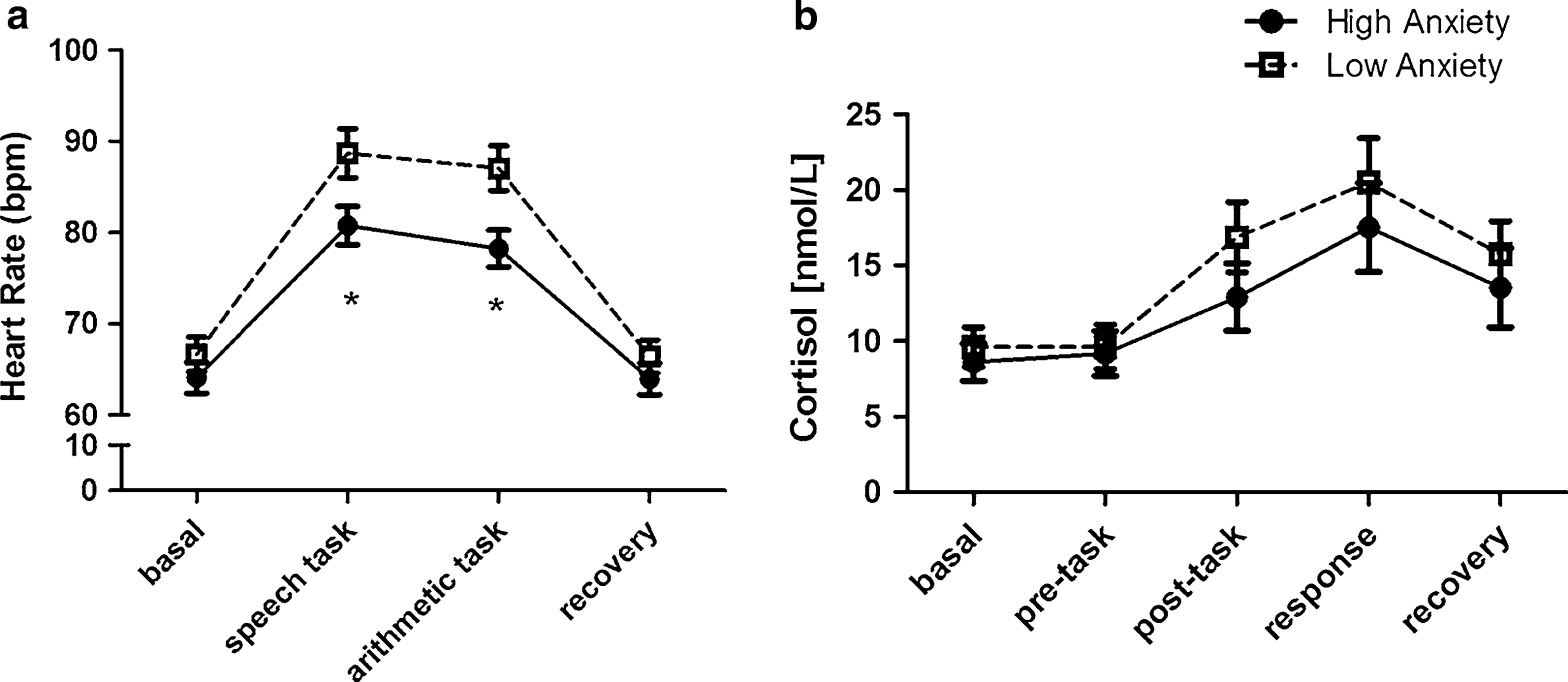
Mean (± SEM) heart rate and cortisol during the TSST for the low anxiety and high anxiety groups. Peacekeepers with a score above the median for trait anxiety are depicted by* circles*, and those with a score below the median are depicted by* squares*. **a** Mean heart rate (± SEM) is depicted at four time points: basal, speech task, arithmetic task and recovery. Participants with higher trait anxiety react less to speech and arithmetic tasks in comparison with those who have lower trait anxiety. *Asterisks* represent a significant difference (p < 0.05) between the groups. **b** Mean salivary cortisol concentration (± SEM) is depicted at five time points relative to the beginning of TSST: −15 min (basal), −5 min (pre-task), +10 min (post-task), +25 min (response) and +40 min (recovery). There was not a significant interaction between conditions and Trait Anxiety

For heart rate, the repeated-measures ANOVA revealed a main effect of CONDITION (F_(3,135)_ = 132.34, p < 0.0001). Pre-planned comparisons showed an increase in heart rate during the speech (Mean = 84.80 bpm) and arithmetic tasks (Mean = 82.72 bpm) compared with the basal level (Mean = 65.39 bpm) (p < 0.0001 for both comparisons). The comparison between the speech and arithmetic tasks did not show a significant difference (p = 0.09). The heart rate during the recovery (Mean = 65.21 bpm) condition was lower than during the speech and arithmetic tasks (p < 0.001 for both comparisons). The heart rate between the recovery and basal conditions did not differ (p = 0.77). TRAIT ANXIETY showed a significant between-subject factor (F_(1,45)_ = 4.84, p = 0.03). Participants with high trait anxiety presented a lower heart rate (Mean = 71.76 bpm) than those with low trait anxiety (Mean = 77.19 bpm). More importantly, there was a significant interaction between CONDITION and TRAIT ANXIETY (F_(3,135)_ = 3.39, p = 0.02). Pre-planned comparisons revealed a significant difference between those with low and high trait anxiety during the speech task (Mean = 88.68 and 80.75 bpm respectively; p = 0.03) and during the arithmetic task (Mean = 87.03 and 78.23 bpm respectively; p = 0.008), indicating that participants with high trait anxiety reacted less than those with low trait anxiety. No significant differences between low and high trait anxiety were found for the other conditions (p > 0.05 for all comparisons) (Fig. 1a). Effect size analyses showed that Cohen’s δ to speech task (low anxiety–high anxiety) was 0.68 and Cohen’s δ to arithmetic task (low anxiety–high anxiety) was 0.81, both representing large effect sizes.

Because the participants with high trait anxiety had higher PCL-C scores than those with low trait anxiety, we conducted an ANCOVA analysis to investigate if having a higher PCL-C score, rather than high trait anxiety, might be responsible for our findings. The ANCOVA analysis showed that having a higher trait anxiety had a significant influence on the results from the different conditions of the experiment (Wilks lambda = 0.78, F_(4,40)_ = 2.75, p = 0.04) but having a higher PCL-C score did not have a significant influence (Wilks lambda = 0.93, F_(4, 40)_ = 0.74, p = 0.57). For cortisol, repeated-measures ANOVA revealed a main effect of CONDITION (F_(4,108)_ = 24.28, p < 0.0001). Pre-planned comparisons showed an increase in cortisol during the post-task (Mean = 14.68 nmol/L) and during response (Mean = 18.45 nmol/L) conditions in comparison with the basal (Mean = 9.05 nmol/L) and pre-task conditions (Mean = 9.37 nmol/L) (p < 0.0001 for all comparisons). The cortisol levels during the recovery (Mean = 14.52 nmol/L) condition were lower than during the response (p < 0.001). There was not a main effect of TRAIT ANXIETY (F_(1,27)_ = 0.60, p = 0.44). In contrast to our observations for heart rate, there was not a significant interaction between CONDITION and TRAIT ANXIETY (F_(4,108)_ = 0.71, p = 0.58) for cortisol analysis (Fig. 1b). This result indicates that trait anxiety did not modulate cortisol response to the stress task.

## Discussion

The present study investigated the influence of trait anxiety on cardiac and cortisol response to an acute psychological stress in healthy Brazilian peacekeepers. Our findings demonstrated a significant increase in heart rate and cortisol in response to the TSST and a significant recovery of both measures in the aftermath. Moreover, peacekeepers with higher trait anxiety showed a reduced heart rate (HR) increase to the acute psychological stress compared to those with lower trait anxiety. This difference was not only significant, but showed a large effect size. Trait anxiety did not modulate cortisol response to the task. Despite the high trait anxiety group having a higher PCL-C score than the low trait anxiety group, it did not influence the cardiac results.

We have previously demonstrated that peacekeepers presenting positive predispositions, with high resting vagal control and/or high resilience trait, showed an allostatic response to stress, that is, a vigorous reaction followed by efficient recovery [31]. In the present study, the reduced responsiveness to stress by anxious peacekeepers may characterize an inadequate response, in line with “Type 2” allostatic overload [32]. As mentioned earlier, a blunted cardiovascular response was proposed by Phillips et al. [6] to be associated with negative health outcomes. Indeed, individuals who experience chronically high levels of anxiety are at an increased risk for several diseases associated with aging, including cardiovascular, autoimmune, and neurodegenerative diseases, as well as for early mortality [33]. Given that anxiety disorders have the highest lifetime prevalence of any psychiatric disorder, with up to 30 % of the population affected during their lifetimes [34], it is important to understand the mechanisms involved in the modulation of the stress response by trait anxiety. There is evidence that across the anxiety disorder spectrum, increased self-reported distress and higher trait anxiety scores are related to decreased physiological reactivity [35, 36]. In the present work, we found a similar result, even in a non-clinical sample. Healthy peacekeepers with above average anxiety trait scores presented less cardiac reactivity.

The differential responsiveness of high and low anxiety sub-groups could reflect different adaptive strategies to cope with stress. It has been suggested that natural selection maintains a balance of different personality traits [37], as exemplified by the “*hawk*-*like*” and “*dove*-*like*” strategies hypothesis. According to the aforementioned study, “*hawks*” were characterized by highly aggressive behavior, preferentially adopting a fight–flight response when establishing a new territory or defending an existing territory. Moreover, they would exhibit low HPA axis responses and high neurosympathetic and adrenomedullary responses to acute threats. On the other hand, “*doves*” were characterized by low levels of aggressive behavior and freeze-hide responses when adapting to threats in their environment. They also had high HPA axis responses as well as low neurosympathetic and adrenomedullary activity to acute threats. Both traits are considered to be adaptive and depend on the environmental conditions. “*Hawks”* are more likely to be violent and more prone to develop impulse control disorders, hypertension, cardiac arrhythmias, sudden death, atypical depression, chronic fatigue states and inflammation, if submitted to allostatic overload. In contrast, under allostatic overload, “*doves”*, are more susceptible to anxiety disorders, metabolic syndromes, melancholic depression, psychotic states and infection. Our measures of the cardiac response to stress may indicate that high-anxiety participants could express “*dove*-*like”* traits and that low-anxiety participants could exhibit “*hawk*-*like”* traits. Both groups may suffer from detrimental effects when exposed to allostatic overload.

In the present study we did not find a significant impact of trait anxiety on cortisol response to an acute stress. Similarly, Bohnen et al. [38] failed to show a significant impact of trait anxiety on cortisol response to a continuous mental task performance. This result indicates that HPA axis phasic activation may not be modulated by this factor. However, it is important to remember that the modulation of the HPA axis in response to stress may vary widely in function in relation to the nature and duration of the stress [24, 39], and it is still possible that trait anxiety may play an important role in modulating cortisol response in other conditions not evaluated here. Nonetheless, considering the main characteristics of the stress task applied here (social evaluation and uncontrollability), the present study was able to reproduce cortisol results, indicating that the adaptation of the TSST for peacekeepers was effective.

Although our study helps to elucidate the role of trait anxiety and cardiac and cortisol reactivity to the TSST, it has some limitations that deserve consideration. The sample size was relatively small, but despite this the magnitude of the differences observed showed large effect sizes. The participants were all military personnel previously exposed to a peacekeeping mission. On the one hand, it was advantageous to study a homogeneous sample composed of only young male adult peacekeepers, but it also prevents a direct generalization of the results to the broader population. Additionally, the measurement of the changes in autonomic function (i.e. heart rate variability and blood pressure variability) associated with some indices of cardiac and vascular function (e.g. cardiac output, stroke volume and peripheral vascular resistance) could have given a better insight into the influence of the autonomic nervous system on the heart rate fluctuation observed. Also, the final analysis of the results could be improved by a self-report assessment of anxiety and stress in response to the TSST. Whether exposure to chronic stress combined with the high anxiety trait is a precondition to blunted reactivity remains an open question.

## Conclusion

We conclude that in a sample of peacekeepers that was homogeneous in terms of gender, age, health and physical conditioning, individuals with higher trait anxiety had less tachycardia in response to acute psychological stress than those with lower trait anxiety. Although we did not find an interaction between the HPA axis and trait anxiety, the reduced cardiac responsiveness to stress in more anxious participants may indicate a blunted inadequate response or a “*dove*-*like*” strategy to cope with stress. The former alternative may argue in favor of the presence of allostatic overload, and the latter can be a warning sign of a risky condition. Regardless of the interpretation, understanding the role of trait anxiety in the stress response may help in the selection, training and treatment of professionals, such as peacekeepers, who are involved in risk-taking activities.

## Methods

### Participants

This study was conducted with 50 male Brazilian Army personnel (privates, corporals, and sergeants) with a mean age of 25.4 years (SD ± 5.99), a mean length of military service of 6.8 years (SD ± 6.28) and a mean body mass index of 23.2 kg/m^2^ (SD ± 4.34), who had volunteered for the United Nations Mission for Stabilization in Haiti (MINUSTAH). They were recruited to take part in the study after responding to an invitation posted in their units. Before embarking on the 6-month peacekeeping mission in Haiti, all participants received the same preparatory instructions and physical training, which was delivered by a specialized training team from the Brazilian Army Physical Fitness Research Institute. The experiments were conducted 4–10 months after returning to Brazil from the peacekeeping mission in Haiti. Participants answered a general questionnaire to verify if they met the inclusion criteria: non-smokers with no reported mental disorders and not taking any medication at the time of the experiment. In addition to the questionnaire, they underwent a clinical interview with a generalist physician before and after the mission.

### Ethics statement

The study was conducted in accordance with the Declaration of Helsinki and was approved by the Ethics Institutional Review Board of the Federal University of Rio de Janeiro. The participants read and signed a statement about ethics, use of data and consent prior to participating in the experiment.

### Psychometric measures

Trait anxiety was assessed with the Brazilian Portuguese version of the STAI-T [6, 40]. This inventory is a 20-item scale for measuring the intensity of anxiety by assessing individual differences in anxiety proneness. The subjects were asked to rate how they feel *in general*. They rated each phrase on a scale of 1–4 (1, *“almost never”* to 4, *“almost always”*). The internal consistency of the Brazilian Portuguese version of STAI-T varies from studies and populations with the Cronbach’s alpha ranging from 0.7 to 0.89 [41].

The Brazilian Portuguese version of the PCL-C [29, 42], was used to investigate posttraumatic stress symptoms. It is considered a screening instrument for detection of probable posttraumatic stress disorder (PTSD) diagnosis. The PCL-C is a 17-item self-report measure of the severity of symptoms in response to a traumatic experience. Using a 5-point Likert scale (1, “*not at all*”, to 5, “*extremely*”), participants rated the extent to which each symptom had affected them in the previous month for each cluster (re-experiencing, avoidance/numbing and hyperarousal), providing a total symptom score (range 17–85). The Brazilian Portuguese version of the PCL-C presents good internal consistency with a Cronbach’s alpha of 0.91 [43].

The MPFSI [44] was developed at the centre for personnel studies for use with Brazilian Army peacekeepers. It is composed of 46 items comprising different stressful situations (e.g. missing one’s family, seeing starving people). For each stressor, the participants were requested to identify if they had experienced it or not. For analysis purposes, we calculated the total number of different stressful situations experienced during the mission by each peacekeeper. The result has high internal consistency (Cronbach’s alpha = 0.90) and satisfactory construct and convergent validity.

### Cardiac measure

One PC-compatible computer was used for the data acquisition of the electrocardiographic parameters and to give instructions to volunteers, using the Acknowledge (BIOPAC Systems Inc.) and Presentation (Neurobehavioral Systems) software programs, respectively. Electrocardiographic recordings were collected at a sampling frequency of 1000 Hz through an electrocardiograph ECG100C module coupled to the MP150 system (BIOPAC Systems Inc.).

An off-line peak detection algorithm (derivative plus threshold) was used to estimate R-wave fiducial points, after which the series was screened by hand and corrected for artifacts. HR was derived from the interval (in milliseconds) between successive R waves and was later converted to beats per minute. Physiological parameters were continuously monitored during the whole session. HR was extracted for the first 2 min of baseline, speech task, arithmetic task and recovery. The first 2 min of the task was used because it has been shown that cardiac reactivity peaks early, when novelty and uncertainty are greatest, and then declines with continuous exposure [45].

Data processing followed the recommendations of the task force of the European Society of Cardiology and the North American Society of Pacing Electrophysiology [46]. We employed KARDIA, a Matlab (MathWorks Inc., MA) software program, for the analysis of cardiac parameters [47, 48].

### Cortisol measure

Saliva samples were collected passively for 5 min, using Salivette^®^. The samples were frozen immediately after collection and were later analyzed by Enzyme Immunoassay (EIA) using DSL-10-671000 ACTIVE^®^ Cortisol Kit. The intra- and inter-assay variation coefficients of variation were <4.8 μg/dL and <7.2 %, respectively. All samples were centrifuged at 1500g for 5 min to obtain a clear supernatant before conducting the EIA analysis.

### Stress task

TSST [21] adapted for peacekeeping troops was used as the stressor [49]. The TSST is a standardized protocol for the induction of moderate to extreme psychological stress in laboratory settings. In this adapted version, the participant had to prepare (10 min) and deliver a speech (5 min) about his qualities as a peacekeeper. He was expected to make a statement about why he would be a good candidate for a future peacekeeping mission. To ensure participant engagement and increase the stress of the task, it was performed in front of two army officers. In cases in which the participant finished his speech ahead of time, the officers discussed it with him until the 5 min period was completed. After the speech, the participant performed an arithmetic task (5 min) in which he was asked to count backwards from 910 by sequentially subtracting 7 (e.g., 910, 903, 896, 889, and so on). After each wrong answer, he was stopped by the officers and had to restart the test from the beginning. The whole session was video recorded, and the participants were informed that the video would be evaluated later.

### Procedure

After completing the written informed consent form, participants sat in front of a table in a sound-attenuated and temperature-controlled (22–24 °C) room. The ECG electrodes were placed on the chest at lead II, after which the participants completed the STAI-T, PCL-C and the MPFSI. After a 20-min period of adaptation to the experimental set-up, a 5-min basal ECG was registered. Next, two army officers entered the room, instructed the volunteer about the speech preparation, and left the room. The participant then had 10 min to prepare his speech. The officers then returned and sat in front of the volunteer to conduct both stress tasks (speech—5 min; arithmetic—5 min). Finally, the participant was instructed to relax for the next 5 min while recovery phase recordings were made. The whole procedure was video recorded. To obtain cortisol measurements, saliva samples were collected at −15, −5, +10, +25 and +40 min in relation to the beginning of the stress task (TSST) and were labelled basal, pre-task, post-task, response and recovery, respectively. For each saliva sample, participants were instructed to rinse their mouth with water and then place a cotton roll (Salivette^®^) under the tongue for 5 min. Participants were instructed not to drink or eat anything 1 h before the beginning of the test. At the end, the participants were thanked and debriefed. To avoid circadian influences, all of the experimental sessions started between 1300 and 1700 h. The experiment was conducted in Brazilian Portuguese.

### Statistics

The heart rate was computed in beats per minute for the first 2 min of each condition of the experiment: basal, speech task, arithmetic task and recovery. Cortisol concentration was described as nmol/L and measured in the following conditions of the experiment: basal, pre-task, post-task, response and recovery. Based on a median-split of trait anxiety score, the participants were assigned to “low” or “high” trait anxiety sub-groups.

Age, body mass index, length of military service, total number of stressful situations, STAI-T and PCL-C scores were compared between “low” and “high” trait anxiety groups with Student’s t test for independent groups.

A mixed design repeated-measures analysis of variance (ANOVA) was performed, with the heart rate as the dependent variable, the CONDITION (basal, speech task, arithmetic task and recovery) as a within-subject factor and TRAIT ANXIETY (low or high) as a between-subject factor. The same model was applied for the analysis between cortisol response and trait anxiety, with cortisol concentration as the dependent variable, the CONDITION (basal, pre-task, post-task, response and recovery) as a within-subject factor and the TRAIT ANXIETY (low or high) as a between-subject factor. Pre-planned ANOVA analyses were used for the investigation of the comparisons of interest.

For heart rate only, we performed two additional analyses. An analysis of covariance (ANCOVA) was performed to investigate the effect of a categorical independent variable (trait anxiety) controlling for the effects of a continuous predictor variable (PCL-C). Effect size measures were estimated using the Cohen’s δ, that converted the means difference between the two groups in the amount of standard deviation. This measure is usually interpreted as follows: <0.20 as small effect, 0.20–0.49 as medium effect and ≥0.50 as large effect.

Data were analyzed using the Statistica 7.0 software program (StatSoft Inc, OK). In all analyses, statistical significance was taken as p < 0.05.
